# Reliability of Shear Wave Elastography for Measuring the Elastic Properties of the Quadratus Lumborum Muscle

**DOI:** 10.3390/diagnostics15060722

**Published:** 2025-03-13

**Authors:** Mónica López-Redondo, Juan Antonio Valera-Calero, Javier Álvarez-González, Alberto Roldán-Ruiz, Sandra Sánchez-Jorge, Jorge Buffet-García, Germán Monclús-Díez, Davinia Vicente-Campos

**Affiliations:** 1 Faculty of Health Sciences, Universidad Francisco de Vitoria, 28223 Madrid, Spain; monica.lopezredondo@ufv.es (M.L.-R.); j.alvarezglez.prof@ufv.es (J.Á.-G.); alberto.roldan@ufv.es (A.R.-R.); j.buffet.prof@ufv.es (J.B.-G.); davinia.vicente@ufv.es (D.V.-C.); 2 Department of Radiology, Rehabilitation and Physiotherapy, Faculty of Nursery, Physiotherapy and Podiatry, Complutense University of Madrid, 28040 Madrid, Spain; gmonclus@ucm.es; 3 Grupo InPhysio, Instituto de Investigación Sanitaria del Hospital Clínico San Carlos (IdISSC), 28040 Madrid, Spain

**Keywords:** low back pain, quadratus lumborum, reliability, reproducibility of results, shear wave elastography

## Abstract

**Background/Objectives**: The quadratus lumborum (QL) muscle is a key structure involved in patients with low back pain (LBP). Since the discriminative capability of morphological descriptors is uncertain and considering the high prevalence of myofascial trigger points and the poor reliability of manual palpation in this condition, developing a reliable procedure for assessing the QL’s tenderness is needed for facilitating the diagnosis and monitoring changes over time. We aimed to analyze the intra- and inter-examiner reliability of SWE for calculating the QL tenderness in patients with LBP. **Methods**: Using a convex transducer, longitudinal shear wave elastography (SWE) images of the QL muscle were acquired bilaterally twice in 52 volunteers with moderate LBP and disability by one experienced examiner and one novel examiner to measure shear wave speed and Young’s modulus as stiffness metrics. **Results**: Intra-examiner reliability estimates demonstrated high consistency independently of the examiner’s experience (intraclass correlation coefficients (ICCs) > 0.930) for both metrics. However, experienced examiners showed smaller minimal detectable changes. Additionally, inter-examiner reliability was lower, with ICCs ranging from 0.57 to 0.68, and significant differences in mean values between examiners (*p* < 0.01) were found. **Conclusions**: This procedure exhibited excellent intra-examiner reliability for assessing QL muscle stiffness in patients suffering LBP, indicating high repeatability of measurements when performed by the same examiner. In addition, experienced examiners demonstrated greater sensitivity in detecting real changes not attributed to measurement errors. However, inter-examiner reliability was moderate, highlighting the need for consistent examiner use to avoid measurement variability and averaging multiple measurements to enhance the accuracy.

## 1. Introduction

The quadratus lumborum (QL) is a muscle located in the posterolateral region of the lumbar area. It attaches to the iliac crest, the transverse processes of the lumbar vertebrae, and the lower edge of the twelfth rib [1]. Although its primary function is dynamic, contributing to lumbar spine extension and lateral flexion, it also serves as an accessory muscle in inspiration [2].

From a clinical perspective, the QL muscle is closely linked to low back pain (LBP), as various functional alterations have been identified. Electromyographic research has highlighted differences in muscle activity distribution between individuals experiencing recurrent LBP and those without symptoms [3,4]. However, anatomical evidence remains inconclusive due to contradictory findings in imaging studies [5]. For instance, Kamaz et al. [6] suggested that chronic LBP leads to muscle atrophy in the QL, whereas Sions, after assessing lumbar levels from the second to the fifth vertebrae, found no correlation between LBP and changes in muscle size or fatty infiltration [7].

Considering that multiple studies have reported a high prevalence of active myofascial trigger points (MTrPs) in patients with non-specific LBP [8,9,10,11,12], assessing muscle stiffness may offer more clinically relevant insights than traditional size, histological, or shape descriptors. MTrPs are defined as hyperirritable, painful spots within taut muscle bands that produce localized and referred pain when mechanically stimulated [13]. Evaluating muscle stiffness can help detect early disease stages, even before morphological abnormalities become visible in standard gray-scale imaging.

In this context, sonoelastography is used to assess tissue elasticity and serves as a valuable complement to conventional B-mode ultrasound imaging (US). While several elastography techniques have been described for musculoskeletal assessment [14], shear wave elastography (SWE) is regarded as the most reliable method. This approach relies on mechanical shear waves, generated by the compressive acoustic waves used in B-mode imaging, to measure shear wave propagation velocity—a key indicator of tissue stiffness.

Since current guidelines stress the importance of evaluating muscle tenderness—particularly in the QL and gluteus medius muscles in individuals with LBP—and given the limited reliability in identifying the presence, quantity, and precise location of MTrPs [10], this study is justified by the need to establish reliable assessment methods. Specifically, developing objective and valid tools to measure muscle elasticity is crucial for improving diagnostic accuracy and clinical evaluation. Developing such a reliable procedure is essential to investigate the accuracy of SWE in further studies to discriminate patients with LBP and asymptomatic subjects and the association between SWE metrics with relevant outcomes recommended in clinical practice guidelines (e.g., range of movement, muscle function, pain intensity, recurrence, chronicity, pain-related disability, pain extent or psychosocial factors) [15]. Consequently, the general aim of this study is to analyze the intra- and inter-examiner reliability of an SWE procedure to examine the stiffness of the QL muscle in a sample of patients with LBP.

## 2. Materials and Methods

### 2.1. Study Design

Between February and May 2024, a longitudinal observational study to determine the inter-examiner reliability estimates of an SWE procedure was conducted in a physiotherapy lab located at a private university in Madrid (Spain). For enhancing the quality of the report, the recommendations declared in the Reporting Reliability and Agreement Studies (GRRAS) guidelines [16] and the Enhancing the QUAlity and Transparency Of health Research (EQUATOR) guidelines [17] were followed. Additionally, the rights of the participants were considered in accordance with the Declaration of Helsinki and a Local Ethics Committee (Universidad Francisco de Vitoria; ID: 15/2024; approval date: 22 February 2024) provided oversight and approval for the study protocol prior to data collection.

### 2.2. Participants

A sample of patients suffering from LBP were recruited by posting local announcements in the physiotherapy clinic and around the campus of Health Sciences. Inclusion criteria included being aged between 18 and 65 years, reporting at least one clinically relevant episode of LBP within the last year, without neurological signs, and reporting at least mild-to-moderate pain intensity (at least 3 points out of 10 in the Visual Analogue Scale [18]) and related disability (at least 12 points out of 100 in the Oswestry Disability Scale [19]) at the data collection point.

Participants were systematically excluded from the study if they met any of the following criteria: use of medications that could affect muscle tone, history of spinal surgery, presence of neuropathies (such as radiculopathies or myelopathies), serious medical conditions (including tumors, fractures, neurological disorders, or systemic diseases), clinically relevant asymmetries, or widespread musculoskeletal disorders like fibromyalgia.

Those who passed the initial screening were provided with a written informed consent form, which they were required to read, understand, and sign before being scheduled for data collection.

### 2.3. Sample Size Calculation

The minimum sample size required for this study was estimated following the instructions provided by Walter et al. [20] for reliability studies using intraclass correlation coefficients (ICCs). Since no previous studies analyzed any intra- or inter-examiner reliability estimates in clinical populations, the results obtained in asymptomatic subjects were used as a reference. Zhou et al. [21] reported ICCs for test–retest reliability ranging between 0.79 and 0.82 in sample of 52 subjects.

Therefore, after setting the minimum acceptable ICC (ρ_0_) at 0.7 (the lower limit of “good reliability” defined in the literature [22]), the expected ICC (ρ_1_) at 0.82, the significance level (α) at 0.05, and the power (1 − β) at 0.80 and considering 2 raters (k) and an expected dropout rate of 10% (due to the longitudinal nature of the study), 36 participants were needed for appropriate statistical power.

### 2.4. Examiners

One experienced examiner with +10 years of experience in musculoskeletal US and clinical experience in the management of patients with musculoskeletal conditions and one novel examiner with less than 1 year of experience in both fields and 20 h of training in US were involved in the study.

To enhance the methodological rigor of the procedures, the imaging acquisition involved the randomization of both the order in which volunteers participated and the initial side examined. The study was divided into two separate sessions: the first morning session took place between 9:00 and 11:00, while the second afternoon session occurred from 13:00 to 15:00 p.m. A strict isolation protocol was implemented, with the two examiners operating on alternating days to prevent any form of communication or agreement between them.

Each participant was required to attend two separate appointments, with a 24 h time interval between each session. Notably, the examiner conducting the assessment alternated between appointments. Later, a third investigator codified all the images. Finally, each rater measured their own images in a randomized sequence, keeping blinded the identity of the participants and side evaluated.

### 2.5. Ultrasound Imaging Acquisition Protocol

The US device used for collecting all the images was a Canon Aplio A device, using a convex transducer 8C1 (Canon Medical Corp, 1385 Shimoishigami, Otawara, Tochigi 324-8550, Japan). The console settings were also standard for all the acquisitions (frequency = 5 MHz, gain = 80 dB, dynamic range = 60 and depth = 12 cm).

**Figure 1:**
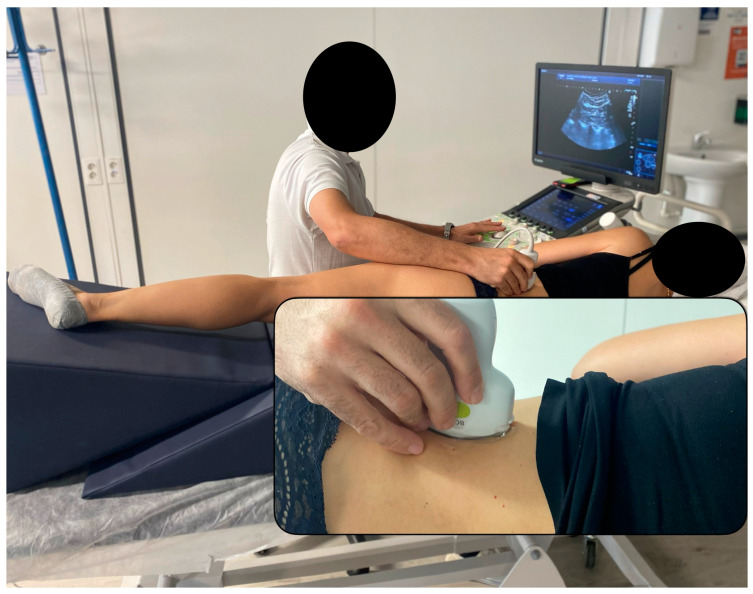
Operator and patient setting for the acquisition of shear wave elastography images of the quadratus lumborum muscle.

The imaging acquisition protocol followed the instructions provided by Zhou et al. [21]. Participants were positioned in a lateral decubitus position to ensure a neutral alignment of both the spine and lower limbs. To facilitate this, a wedge-shaped cushion was placed behind the upper thoracic region, helping to maintain the torso at a right angle to the examination table. Additionally, a square cushion was positioned between the thighs to preserve hip joint neutrality. If required, a towel was used to support the lumbar spine and maintain its natural alignment (Figure 1). To prevent any morphological distortions due to muscle contractions, participants were instructed to remain completely relaxed throughout the procedure [23].

Once acoustic coupling gel was applied to the transducer; it was initially positioned just above the iliac crest along the mid-axillary line. To optimize imaging, the cranial end of the transducer was tilted approximately 20° posteriorly, allowing the L4 vertebra to be centered within the B-mode ultrasound field. At this location, the QL appeared as a relatively hypoechoic muscle situated above the psoas major, which was identified as the muscle covering the vertebral bodies (Figure 1).

**Figure 2:**
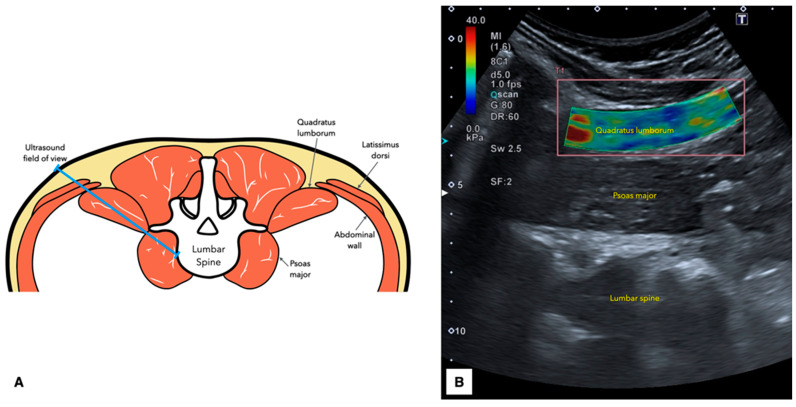
Anatomical cross-sectional illustration of the quadratus lumborum identifying the probe field of view (**A**) and an illustrative example of shear wave elastography image (**B**).

To capture the QL images, the transducer’s orientation was firstly fine-tuned to be aligned parallel to the long axis of the muscle fibers, ensuring perpendicularity of the central portion of the transducer to the muscle fibers. Once this procedure was completed, the SWE mode was enabled (placing the region of interest on the center of the muscle and covering at least a 50% of the muscle) to save the image. Finally, the images were measured by contouring a freely drawn quantification box, ensuring that it did not overlap the edges and it covered the muscle excluding the muscle fasciae (Figure 2).

### 2.6. Statistical Analysis

All data processing and analyses were conducted in the Statistical Package for the Social Sciences (SPSS) v.29.1.1 (Armonk, NY, USA) for Mac OS. All tests were two-tailed, and the significance level cut-off was set at *p* < 0.05. Initially, the distribution of continuous variables was assessed by employing histograms and Shapiro–Wilk tests.

Next, the demographic and clinical characteristics of the sample were summarized using descriptive statistics. For categorical variables, the distribution was presented as frequencies and percentages, including the number and proportion of male and female participants. On the other hand, continuous variables were described using central tendency (mean or median) and dispersion (standard deviation or interquartile range) metrics depending on whether they followed a normal distribution. Gender differences were assessed by using Student’s *T*-tests for independent samples (providing the mean difference, 95% Confidence Interval, and *p* value).

For the reliability analyses, the following calculations were performed: (1) the mean average and standard deviation of both operators for each US metric; (2) measurement disagreement between examiners (calculated as the difference between trials for intra-examiner reliability and the difference between examiners with single measures and an average of 2 attempts for inter-examiner reliability, providing also the absolute error to avoid errors underestimation); (3) the intraclass correlation coefficients (ICC_3,1_ for intra-examiner reliability and ICC_3,2_ for inter-examiner reliability were both assessed using a 2-way mixed model with a consistency type); (4) the standard error of measurement (SEM was determined by multiplying the standard deviation of the mean by the square root of 1 minus the ICC); and (5) the minimal detectable changes (MDCs, computed as 1.96 times the square root of 2 times the SEM) [22].

## 3. Results

During the recruitment period, 52 individuals expressed interest in participating in the study. Five participants (*n* = 5) were excluded from analyses as they did not reach the minimum cut-off scores for pain intensity (*n* = 3), disability (*n* = 1) or both (*n* = 1). Additionally, all acquired images were valid and analyzed, resulting in no data loss. Consequently, the study included *n* = 31 females and *n* = 16 males, with *n* = 94 images taken of the QL muscle from both the left and right sides. Since each examiner acquired 2 images per muscle, a total of 376 SWE images were acquired in this study.

Table 1 summarizes the demographic and clinical characteristics of the sample included in the study, comparing males and females. The demographic data showed significant differences between genders. Males were significantly younger (*p* < 0.001), heavier (*p* < 0.001) and taller (*p* < 0.001) compared to females. Additionally, significant body composition differences were found. Males had a higher BMI (*p* = 0.009) and higher water volume (*p* < 0.001) compared to females. Despite the BMI differences, there was no significant difference in body fat percentage between genders (*p* = 0.271). Regarding the clinical characteristics, both genders presented comparable pain intensity (moderate pain intensity, *p* = 0.584) and pain-related disability (moderate disability, *p* = 0.209).

Test–retest reliability estimates to determine QL stiffness are detailed in Table 2. No statistically significant differences were found between Trial 1 and Trial 2 for either the experienced or novice examiners in the assessment of shear wave speed and Young’s modulus (both *p* > 0.05). Although the experienced examiner obtained slightly better ICCs compared to the novel examiner in the assessment (shear wave speed: ICC = 0.954 to 0.979 and ICC = 0.903 to 0.956, respectively; Young’s modulus: ICC = 0.970;0.986 and ICC = 0.965 to 0.984, respectively), both examiners showed excellent test–retest reliability. However, experienced examiners demonstrated greater ability to detect real changes (not attributed to measurement errors) as MDCs were smaller in both metrics in comparison with the novice examiner.

**Figure 3:**
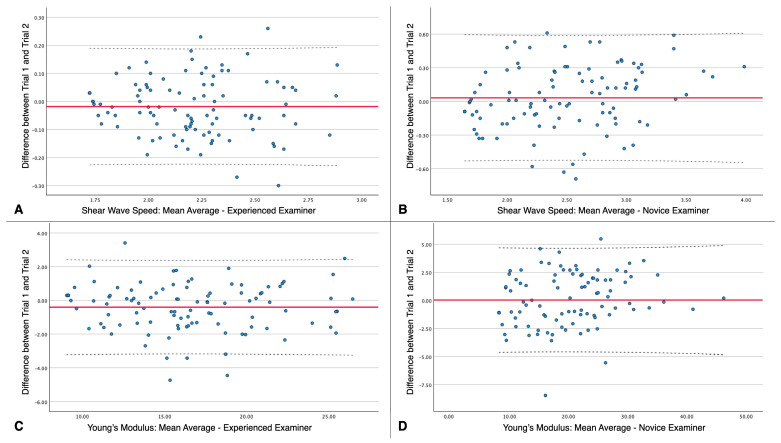
Bland–Altman plots illustrating the test–retest reliability of shear wave speed (SWS) and Young’s modulus (YM) measurements for experienced and novice examiners. (**A**) SWS measurements by the experienced examiner, (**B**) SWS measurements by the novice examiner, (**C**) YM measurements by the experienced examiner, and (**D**) YM measurements by the novice examiner. The plots depict the difference between Trial 1 and Trial 2 (*Y*-axis) against the mean values of both trials for each examiner (*X*-axis). The red line represents the mean difference, while the dashed lines indicate the limits of agreement.

Bland–Altman plots illustrate the test–retest reliability by indicating the mean scores of both trials for each examiner in the *X*-axis and the difference between trials in the *Y*-axis (Figure 3).

Estimates for inter-examiner reliability are detailed in Table 3. Mean difference analyses revealed that the scores obtained by both examiners were significantly different, independently if single and mean average measures are compared (shear wave speed: single measurement and mean average of two measurements *p* = 0.001; Young’s modulus: single measurement and mean average of two measurements *p* < 0.01). In accordance with mean score differences, the ICC scores obtained for inter-examiner reliability were considerably lower in comparison with the ICCs obtained for intra-examiner reliability. Single measurements resulted in moderate inter-examiner reliability (shear wave speed: ICC = 0.571 (0.367;0.710); Young’s modulus: ICC = 0.589 (0.392;0.722)). ICC scores for the mean average of two measurements were moderate, but higher (shear wave speed: ICC = 0.682 (0.530;0.785); Young’s modulus: ICC = 0.648 (0.480;0.762)). Since ICCs were higher for a mean average of two measurements, the MDCs obtained were accordingly lower for the mean average of two measurements.

## 4. Discussion

This is the first study assessing the intra- and inter-examiner reliability of SWE to evaluate the stiffness of the QL muscle in patients suffering LBP. One of the most relevant findings was that test–retest reliability for both experienced and novice examiners showed high consistency, as evidenced by the near-perfect ICC values (all metrics, ICC > 0.93). This suggests that when the same examiner performs the SWE measurements on the QL muscle in patients with LBP, the Young’s modulus and shear wave speed results are highly repeatable. However, the inter-examiner reliability presented a different picture. Shear wave speed and Young’s modulus values varied significantly between experienced and novice examiners, as indicated by the significant mean differences, the higher error, absolute error, SEM and MDC values, and the lower ICCs obtained. This disparity highlights that while individual examiners, regardless of experience level, can reliably reproduce their own measurements, there is a notable discrepancy when different examiners perform the measurements.

This inter-examiner variability may be attributed to flaws regarding specific transducer factors (such as different applied pressure, positioning/orientation, or tilting) or contouring interpretation of the SWE images (since previous reports have described various histological changes linked to chronic pain [24,25,26,27,28,29] which may complicate achieving a clear muscle contour [30]). Thus, variations in individual muscle properties among participants, such as subtle anatomical differences, local muscle stiffness heterogeneity, and potential changes in tissue behavior due to postural adaptations or muscle guarding, may have contributed to discrepancies between examiners. Given that these factors can differentially affect less-experienced examiners, the wider standard deviations obtained by the novice examiner and the errors between trials further suggest that experience plays a crucial role in minimizing measurement variability. However, it is important to highlight that the intra-examiner reliability remained excellent regardless of the examiner’s experience, supporting the accuracy and repeatability of the procedure when conducted by a single operator. Since poorer inter-examiner ICCs (in comparison with intra-examiner) were hypothesized, single measurements and the mean average of two measurements were analyzed separately. The reliability estimates for single measurements versus the mean average of two measurements indicate that averaging multiple trials improves reliability and accuracy, in accordance with many other procedures described in the literature [31,32,33].

Although the main novelty of this study is the inclusion of a clinical population, this is not the first study analyzing the SWE reliability for this muscle as recognized in the introduction. The reliability estimates observed in our study and the study by Zhou et al. [21] revealed both similarities and differences in SWE measurements for muscle stiffness. While our study found near-perfect intra-examiner reliability for the QL muscle with ICC values greater than 0.93, Zhou et al. [21] reported slightly lower but still high reliability for the QL with ICC values ranging from 0.79 to 0.82. This difference could be due to variations in participant characteristics, slight muscle assessment protocols, or examiner experience.

Zhou et al. [21] investigated the influence of gender and physical activity on muscle stiffness, highlighting significant differences. Their findings indicated that QL stiffness was greater in females and those with lower levels of physical activity. These gender-related differences in muscle stiffness may be explained by variations in muscle composition, as females generally have a higher fat percentage and a distinct distribution of muscle fiber types compared to males. These findings highlight the importance of considering demographic and lifestyle factors when interpreting SWE measurements, as they can significantly influence muscle stiffness outcomes and explain the score differences across both studies.

Based on our results for subjects suffering LBP, three recommendations can be provided. Our first recommendation is to ensure that both in research and clinical settings, the same operator should always perform the interpretation and acquisition of the SWE images. This is crucial because involving multiple operators can lead to inconsistencies and misinterpretations, attributing changes in muscle stiffness that are not real but rather due to differences in technique and measurement variability. The significant discrepancies observed in inter-examiner reliability underscore the importance of this recommendation.

Secondly, in situations where it is necessary to involve more than one examiner, it is advisable to calculate the average of at least two measurements to reduce errors and improve consistency. Averaging helps to mitigate the effects of individual examiner variability and enhances the overall accuracy of the measurements, making it a practical strategy for studies and clinical assessments that require multiple operators.

Finally, in longitudinal analyses, where follow-up assessments are conducted over time, it is particularly important to involve an experienced examiner. The lower MDC values observed in experienced examiners indicate that they are more precise in detecting true changes in muscle stiffness. This precision is crucial for accurately determining whether changes in the QL muscle stiffness over time are due to actual physiological changes or merely measurement errors.

### Strenghts and Limitations

Despite the significant findings and novelty of this study (as the first study assessing the intra- and inter-examiner reliability of SWE to evaluate the stiffness of the QL muscle in patients suffering LBP), several limitations should be acknowledged. First, our sample was restricted to patients with mechanical LBP. Given the various classifications of LBP, including cases with mobility deficits, radiating pain, movement coordination impairments, and central sensitization, the stiffness characteristics of the QL muscle may differ depending on the pain etiology. Further studies should explore these subgroups to confirm the applicability of our results across different clinical presentations.

Second, although the minimum sample size to achieve statistically acceptable power was reached, the sample was not sufficiently heterogeneous to allow for definitive conclusions. Future research should include participants with a broader range of clinical severity, particularly those with higher pain-related disability and central sensitization, as these factors could influence muscle stiffness. Additionally, side-specific analyses may provide further insights into potential asymmetries in muscle stiffness.

Third, while the SWE protocol demonstrated excellent intra-examiner reliability, inter-examiner variability remained a concern. Our study employed a single SWE device, which may limit the generalizability of the findings. Additional research incorporating multiple examiners with varying experience levels and different US devices is required to enhance external validity.

Lastly, LBP is a complex condition influenced by a wide range of factors, including psychological, biomechanical, nociceptive, behavioral, environmental, social, and occupational elements. Future studies should focus on examining additional variables, such as psychological stress and anxiety, routinary physical activity at work or during leisure time, and lifestyle patterns, as these may act as confounding factors in muscle stiffness assessments. Gaining deeper insights into these influences will be essential for enhancing the clinical relevance of shear wave elastography in evaluating muscle changes associated with LBP.

## 5. Conclusions

This study evaluated the intra- and inter-examiner reliability of SWE for measuring the stiffness of the QL muscle in patients with chronic LBP. The results indicated high test–retest reliability for both experienced and novice examiners, with near-perfect intraclass correlation coefficients (ICCs > 0.93). This suggests that SWE measurements of the QL muscle are highly repeatable when performed by the same examiner.

However, inter-examiner reliability was notably lower, with significant variability in both SWE metrics between the experienced and novice examiners. Therefore, the most important limitation of this procedure is the need for consistent examiner use to avoid errors. If multiple examiners are necessary, averaging multiple measurements is recommended to improve the scores’ accuracy.

Failure to adhere to these recommendations could lead to significant inconsistencies in muscle stiffness assessments, potentially resulting in misinterpretation of changes in patient status over time. In clinical settings, this may lead to inappropriate treatment decisions, either by overestimating or underestimating true physiological changes in the QL muscle. Similarly, in research contexts, unreliable data could compromise the validity of findings, limiting the ability to draw meaningful conclusions about the relationship between muscle stiffness and LBP. Standardizing examiner protocols and measurement techniques is essential to ensure accurate and clinically meaningful SWE assessments.

## Figures and Tables

**Table 1:** Demographic and clinical characteristics of the sample recruited.

Variables	Subjects with Low Back Pain (*n* = 47)		Difference (95% CI)
	Females (*n* = 31)	Males (*n* = 16)	
Demographics			
Age, years	32.5 ± 13.4	24.6 ± 7.7	7.9 (0.6;15.3) *p* < 0.001
Weight, kg	69.9 ± 13.6	92.8 ± 13.8	22.9 (14.3;31.4) *p* < 0.001
Height, m	1.65 ± 0.04	1.76 ± 0.08	0.11 (0.08;0.15) *p* < 0.001
BMI, kg/m^2^	25.7 ± 5.1	29.9 ± 4.9	4.2 (1.1;7.3) *p* = 0.009
Water volume, L	32.3 ± 3.17	48.5 ± 8.8	16.1 (10.6;21.6) *p* < 0.001
Body fat, %	33.0 ± 10.5	25.4 ± 12.2	7.6 (−6.5;21.7) *p* = 0.271
Clinical Characteristics			
VAS, 0–10	4.9 ± 1.7	5.2 ± 1.9	0.3 (−0.8;1.4); *p* = 0.584
ODI, 0–100	24.8 ± 9.2	25.3 ± 9.0	0.5 (−0.2;1.3) *p* = 0.209

Abbreviations: CI: Confidence Interval; ODI: Oswestry Disability Index; VAS: Visual Analogue Scale.

**Table 2:** Test–retest reliability estimates to determine quadratus lumborum stiffness.

Reliability Estimates	Single Measurement (Trial 1)	Mean Average of 2 Measurements (Average of Trial 1 and 2)
Shear Wave Speed (m/s)		
Mean	2.34 ± 0.33	2.33 ± 0.31
Error	0.30 ± 0.51	0.28 ± 0.42
Absolute Error	0.45 ± 0.39	0.37 ± 0.34
ICC	0.571 (0.367;0.710)	0.682 (0.530;0.785)
SEM	0.22	0.18
MDC	0.60	0.49
Young’s Modulus (kPa)		
Mean	17.75 ± 4.62	17.84 ± 4.55
Error	2.74 ± 7.58	2.87 ± 6.90
Absolute Error	6.65 ± 4.50	6.06 ± 4.32
ICC	0.589 (0.392;0.722)	0.648 (0.480;0.762)
SEM	3.0	2.7
MDC	8.2	7.5

Abbreviations: ICC: intraclass correlation coefficient; MDC: minimal detectable change; SEM: standard error of measurement.

**Table 3:** Inter-examiner reliability analysis: single and mean average measure scores to determine quadratus lumborum stiffness.

Reliability Estimates	Experienced Examiner		Novice Examiner	
	Trial 1 (*n* = 104)	Trial 2 (*n* = 104)	Trial 1 (*n* = 104)	Trial 2 (*n* = 104)
Shear Wave Speed (m/s)				
Mean	2.18 ± 0.29	2.20 ± 0.29	2.49 ± 0.59	2.46 ± 0.52
Error	−0.01 ± 0.10		0.03 ± 0.27	
Absolute Error	0.08 ± 0.06		0.22 ± 0.16	
ICC	0.969 (0.954;0.979)		0.934 (0.903;0.956)	
SEM	0.05		0.13	
MDC	0.14		0.37	
Young’s Modulus (kPa)				
Mean	16.21 ± 4.56	16.61 ± 4.63	19.29 ± 8.44	19.26 ± 7.93
Error	−0.40 ± 1.39		0.03 ± 2.31	
Absolute Error	1.08 ± 0.95		1.91 ± 1.29	
ICC	0.980 (0.970;0.986)		0.976 (0.965;0.984)	
SEM	0.64		1.22	
MDC	1.78		3.40	

Abbreviations: ICC: intraclass correlation coefficient; MDC: minimal detectable change; SEM: standard error of measurement.

## Data Availability

The raw data supporting the conclusions of this article will be made available by the authors on request.
